# The Ripple Effect of Graphite Nanofilm on Stretchable Polydimethylsiloxane for Optical Sensing

**DOI:** 10.3390/nano11112934

**Published:** 2021-11-02

**Authors:** Kossi A. A. Min-Dianey, Top Khac Le, Akeel Qadir, Noé Landry Privace M’Bouana, Muhammad Malik, Sok Won Kim, Jeong Ryeol Choi, Phuong V. Pham

**Affiliations:** 1 Département de Physique, Faculté Des Sciences (FDS), Université de Lomé, Lomé 01BP1515, Togo; anyaratt20@yahoo.fr; 2 Department of Physics and Energy Harvest Storage Research Center, University of Ulsan, Ulsan 44610, Korea; lekhactop@gmail.com; 3 Research Center of Smart Sensing Chips, Ningbo Institute of Northwestern Polytechnical University, Ningbo 315103, China; aki@nwpu.edu.cn; 4 Key Laboratory of Micro/Nano Systems for Aerospace (Ministry of Education), and Shaanxi Province Key Laboratory of Micro and Nano Electro-Mechanical Systems, Department of Microsystems Engineering, Northwestern Polytechnical University, Xi’an 710072, China; 5 Institut Supérieur de Technologie, Université de Bangui, Bangui BP 892, Central African Republic; mbouana@hotmail.fr; 6 Department of Electrical Engineering and Technology, Government College University, Faisalabad 38000, Pakistan; mmalik@gcuf.edu.pk; 7 Department of Nanoengineering, Kyonggi University, Suwon 16227, Korea; 8 SKKU Advanced Institute of Nano Technology, Sungkyunkwan University, Suwon 440746, Korea

**Keywords:** nanofilm, rippled graphite, PDMS, stretchable, optical sensing, photoresponsivity

## Abstract

Graphene-based optical sensing devices have been widely studied for their broad band absorption, high carrier mobility, and mechanical flexibility. Due to graphene’s weak light absorption, studies on graphene-based optical sensing thus far have focused on hybrid heterostructure devices to enhance photo-absorption. Such hybrid devices need a complicated integration process and lead to deteriorating carrier mobility as a result of heterogeneous interfaces. Rippled or wrinkled graphene has been studied in electronic and optoelectronic devices. However, concrete demonstrations of the impact of the morphology of nanofilms (e.g., graphite and graphene) associated with light absorption in optical sensing devices have not been fully examined. This study explored the optical sensing potential of a graphite nanofilm surface with ripples induced by a stretchable polydimethylsiloxane (PDMS) supporting layer under different stretch:release ratios and then transferred onto silicon, both under experimental conditions and via simulation. The optical sensing potential of the rippled graphite nanofilm was significantly enhanced (260 mA/W at the stretch–release state of 30%), as compared to the pristine graphite/PDMS (20 mA/W at the stretch–release state of 0%) under laser illumination at a wavelength of 532 nm. In addition, the results of our simulated computation also confirmed the improved light absorption of rippled graphite nanofilm surface-based optical sensing devices, which was comparable with the results found in the experiment.

## 1. Introduction

Single-layer graphite, or graphene, is a flat hexagonal carbon structure with remarkable properties such as high mobility, high transparency, and excellent conductivity [1,2,3,4,5,6,7,8,9,10,11,12]. It also exhibits potential as a material for detecting molecules, which would offer ultimate sensitivity for gas sensors [13,14], pressure sensors [15,16], photodetectors [17,18,19], biosensors [20], strain sensors [21], and temperature sensors [22]. The intrinsic nature of graphene exhibits weak optical absorption (2.3%) within the visible wavelength range (400–700 nm) [23]; therefore, investigating methods to enhance its absorption are critical for its potential applications.

Recently, graphene with a crumpled (or rough) shape has been utilized widely in electronics [4], energy storage [24,25], composites [26,27], and biomedicine [28]. Although the bendable degree of crumpling affects graphene’s features and the device performance [27,29,30], the understanding of wrinkled or rippled graphene and graphite nanofilms on optical sensing is not yet fully understood. The crumpling of graphene has been investigated in stretched polymers such as polydimethylsiloxane (PDMS) (i.e., high-performance bonding tape) and Ecoflex [31,32]. Zang et al. reported a technique to manipulate the crumpling and unfolding of large-area graphene placed on uniaxially or biaxially relaxed PDMS [31]. By controlling pre-strain relaxation, graphene could be crumpled into a tailored hierarchical structure. Moreover, this crumpled graphene–polymer laminate could lead to the design and/or improvement of conductive coatings, superhydrophobic electrodes, and artificial muscle actuators [31]. In 2016, Kang et al., examined VHB- and Ecoflex-based stretchable optical sensing devices based on crumpled graphene, which exhibited enhanced photoresponsivity [32]. Moreover, simulated computation using the finite element method was performed to prove that crumpled graphene at the edge site of a device showed higher optical absorptivity than at other sites on the visible wavelength [33]. Although the above reports have systematically applied crumpled graphene in different scenarios in an effort to observe any enhancement in light absorption, the effects of rippled graphite (or multilayer, rippled graphene) created via stretchable supporting layers (e.g., PDMS) under different stretch:release ratios have not yet been established and require further study.

Here, we present the optical sensing potential of rippled graphite nanofilms at a wavelength of 532 nm (under experimental conditions) and in a wavelength range of 400–800 nm (via simulation). Consequently, we obtained enhanced photoresponsivity by improving the ripple density of the graphite nanofilm surfaces assisted by a supporting PDMS layer.

## 2. Materials and Methods

Material Preparation: The intrinsic graphite (~12 nm thick, ~36 layers) was manufactured similarly to in previous reports by Sone et al. [34] and Min-Dianey et al. [35], through the exfoliation of a highly oriented pyrolytic nanofilm crystal micro sheet.

Device Processing: The pristine and rippled graphite nanofilms were transferred onto the etched Si window of an optical sensing device. The optical sensing devices were manufactured on a SiO_2_ layer. (i) The SiO_2_ layer was patterned with UV lithography. Electron-beam deposition processes were utilized to deposit Au/Ti electrodes (60/4 nm thick) as contact pads onto the SiO_2_ layer. (ii) Photolithography was utilized to pattern the window (5 × 5 mm^2^). Then, the SiO_2_ layer on the window was etched away by a buffered oxide etchant to form the Si window. (iii) To form a rippled graphite nanofilm/Si Schottky junction, the rippled graphite nanofilm was transferred by flipping the rippled graphite/stretched PDMS specimens upside-down on top of the etched Si window. (iv) Ohmic contact was formed by using Ga–In paste on the back of the SiO_2_ substrate; finally, Au wire was bonded to the top of the electrodes.

Characterization: The UV–Vis spectra (Shimadzu-3600, Shimadzu Corp., Tokyo, Japan) were utilized to measure the optical transparency of the device. Raman microscopy (RM-1000 Invia, Renishaw plc., Wolton-under-Edge, Gloucestershire, UK) was utilized for analysis of the graphite nanofilm. FE-SEM apparatus (Hitachi S-4700, Michigan Tech., Houghton, Michigan, USA) was utilized to capture the surface morphology for the four cases of the graphite nanofilm surfaces under different stretch:release ratios of the PDMS supporting layer (0%, 10%, 30%, and 50%). An atomic force microscope (AFM, Bruker Corporation, Billerica, MA, USA) was utilized to analyze the roughness of the graphite/PDMS surface under the different PDMS stretch:release ratios. I–V curves were determined with an Agilent Semiconductor Analyzer (B1500, Keysight Technology Comp., Culver city, CA, USA) using a wavelength of 532 nm for the rippled graphite nanofilm-based optical sensing devices under different PDMS stretch:release ratios.

## 3. Results and Discussion

**Figure 1:**
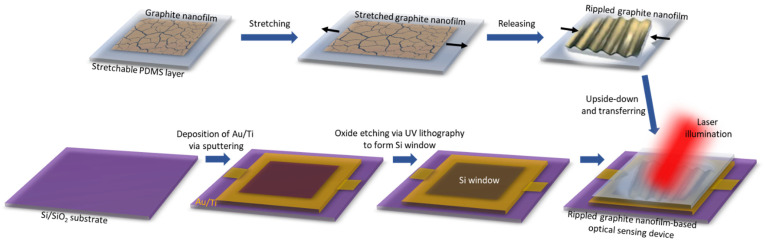
Fabrication process of rippled graphite nanofilm-based optical sensing device.

**Figure 2:**
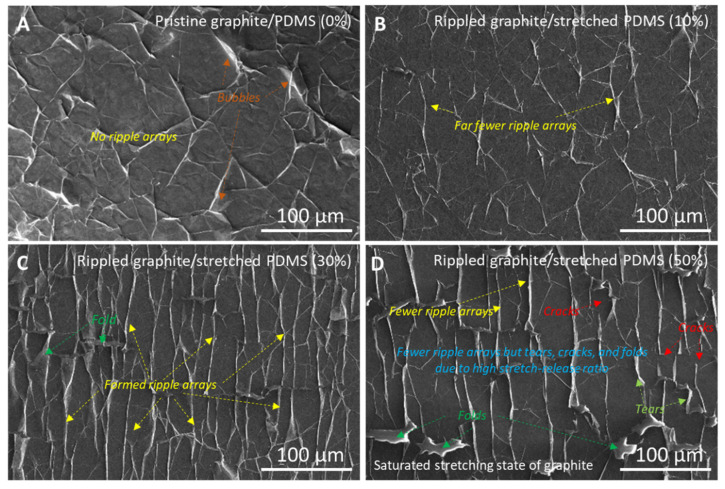
SEM images of (**A**) pristine graphite/PDMS (0%), (**B**) rippled graphite/stretched PDMS (10%), (**C**) rippled graphite/stretched PDMS (30%), and (**D**) rippled graphite/stretched PDMS (50%).

Figure 1 depicts the fabrication process of a rippled graphite nanofilm-based optical sensing device using a PDMS supporting layer for the stretching and releasing of the graphite nanofilm before transferring it onto the aforementioned Si window (see the Section 2 for details). The stretch–release was performed on a PDMS substrate. PDMS proved to be an excellent substrate on which to study the optical properties of graphene. For instance, the optical out-of-plane susceptibility of graphene was studied by the application of graphene onto the PDMS layer [36]. Figure 2 shows field-emission scanning electron microscopy (FE-SEM) images of the morphology of the graphite nanofilm surfaces that were fabricated similarly to previous studies [34,35]. Here, the graphite nanofilm was created with a thickness of 12 nm and was stretched under different stretch:release ratios (0% (pristine), 10%, 30%, and 50%) of the PDMS supporting layer, which was 2 cm in length and 4 mm thick. Specialized apparatus was utilized to create the stretching–releasing effect of the graphite nanofilms on the PDMS supporting layer (Figures S1 and S2). Figure 2A shows an SEM image of pristine (0%) graphite/PDMS with no ripple arrays, but with a few graphite bubbles on the surface due to the slightly rough surface of the PDMS layer (0.9 nm) (Figure S3); meanwhile, the ripple effect of the graphite nanofilm was formed with a few ripple arrays after a stretch–release of 10% of the PDMS length via x-axis movement (Figure 2B). In Figure 2C and Figure S4A, the highly ordered ripple arrays (~20 µm height, ~12 nm thick) of the graphite nanofilm were formed after a stretch:release ratio of 30%, considered the optimized condition for optical sensing. In addition, when we applied a higher stretch:release ratio of 50% (Figure 2D and Figure S4B), the surface of the graphite had fewer ripple arrays as well as more cracks, tears, and folds. This confirmed that the ratio of 50% was the saturated stretching state of the graphite nanofilm.

**Figure 3:**
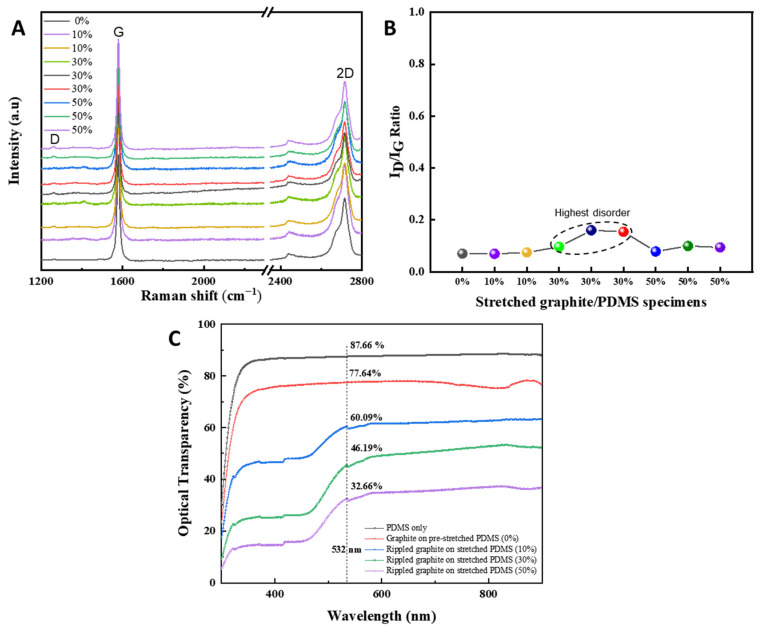
(**A**) Raman spectra of multiple specimens of graphite/PDMS under different stretch:release ratios. (**B**) I_D_:I_G_ ratios of graphite/PDMS specimens in (**A**). (**C**) Optical transparency of graphite/PDMS specimens under different stretch:release ratios (0%, 10%, 30%, and 50%).

To analyze the multilayer phase and the disorder of the graphite nanofilms under different stretch:release ratios (0%, 10%, 30%, and 50%), a Raman microscope was used (Figure 3A,B). The data showed the multilayer structures of the graphite nanofilms, and among those, the rippled graphite on a stretched PDMS layer (30%) exhibited the highest disorder in the graphite structure as the formation of graphite ripples corresponded to the highest roughness of 1.53 µm in an atomic force microscope (AFM) (Figure S3G,H). To visualize the optical transparency characteristics of PDMS, graphite/PDMS (0%), rippled graphite/stretched PDMS (10%), rippled graphite/stretched PDMS (30%), rippled graphite/stretched PDMS (50%), and the ultraviolet–visible (UV–Vis) spectra were captured (Figure 3C). The optical transparency values were 87.66%, 77.64%, 60.09%, 46.19%, and 32.66%, respectively.

**Figure 4:**
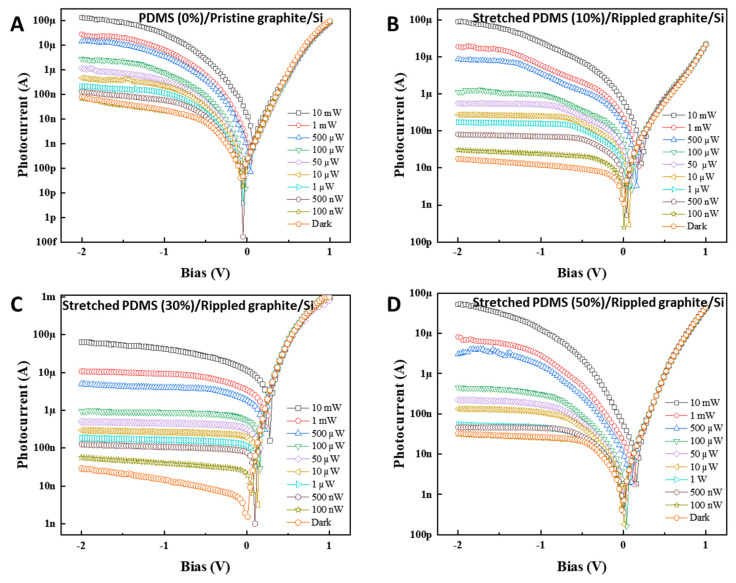
I–V curves of optical sensing devices based on (**A**) PDMS (0%)/pristine graphite/Si, (**B**) stretched PDMS (10%)/rippled graphite/Si, (**C**) stretched PDMS (30%)/rippled graphite/Si, and (**D**) stretched PDMS (50%)/rippled graphite/Si under dark and laser illumination at 532 nm wavelength with different power values.

To understand the optical sensing potential of rippled graphite nanofilm-based devices, we carried out the experiments under dark and laser illumination conditions at a wavelength of 532 nm with different power values (Figure 4). I–V curves were traced for each state: PDMS (0%)/pristine graphite/Si, stretched PDMS (10%)/ rippled graphite/Si, stretched PDMS (30%)/rippled graphite/Si, and stretched PDMS (50%)/rippled graphite/Si. Figure 4A–D shows the dark current (I_dark_) and the photocurrent (I_light_) as functions of bias voltage (V), varying from −2 to 1 V with an exposure under 532 nm visible light with different incident powers (P_incident_) from 100 nW to 10 mW. As the laser power intensity increased, the photocurrent also increased along a reverse bias of −2 to 0. In the optoelectronic field, photoresponsivity [R = (I_light_-I_dark_)/P_incident_] is one of the most important factors to assess the optical sensing potential of the device. As summarized in Table 1, the photoresponsivity (R) and noise-equivalent power (NEP) values for the four aforementioned cases converted at a reverse bias of −1 V (Figure 4A–D) as a function of the various laser power intensities. As the result, the R value of the rippled surface at 30% was the highest, and this was achieved at 260 mA/W, which was 13 times larger than the result found for the pristine graphite surface (20 mA/W). This clearly confirmed that the crumple (or wrinkle) effect [30,32,33] on the nanofilm enhanced its light absorption. Conversely, the NEP values were calculated by $NEP=\frac{\sqrt{2e.I_{dark}}}{R.\sqrt{A}}$ (with A as the effective area (5 × 5 mm^2^)) of rippled and pristine graphite surfaces. As a result, the NEP values were 15.74 × 10^−9^ W/(Hz)^1/2^, 9.65 × 10^−9^ W/(Hz)^1/2^, 7.54 × 10^−9^ W/(Hz)^1/2^, and 10.21 × 10^−9^ W/(Hz)^1/2^, corresponding to the stretch:release ratios of 0%, 10%, 30%, and 50%, respectively. Among those, the NEP of the rippled surface at 30% showed the lowest value (7.54 × 10^−9^ W/(Hz)^1/2^), which was clear evidence that this was the optimal condition. This was a pioneering study in this field and had not previously been studied in any report.

Table 1 summarizes the highest photoresponsivity values of each stretch:release ratio (0%, 10%, 30%, and 50%) of the graphite/PDMS specimens at the lowest power (100 nW) under 532 nm laser illumination. The results were 20 mA/W, 120 mA/W, 260 mA/W, and 13 mA/W, which corresponded to ratios of 0%, 10%, 30%, and 50%, respectively.

**Figure 5:**
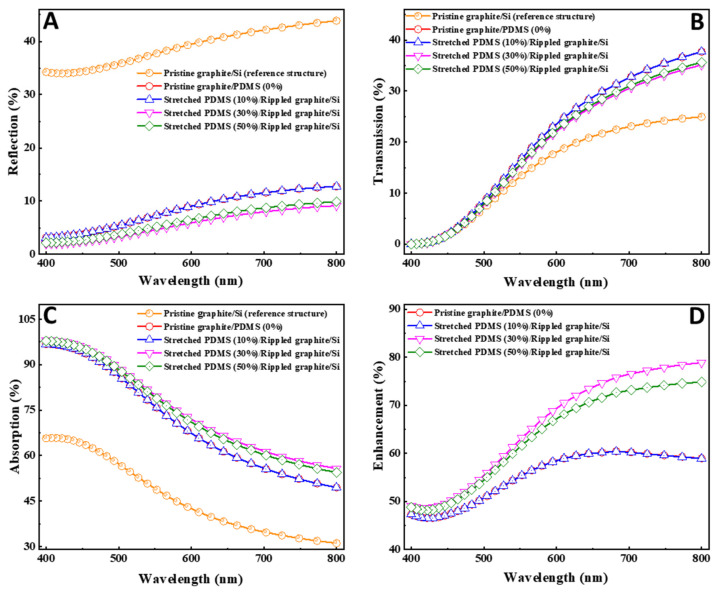
Lumerical finite-difference time-domain (FDTD) computation for the optical characteristics of the spectra of (**A**) reflection, (**B**) transmission, (**C**) absorption, and (**D**) light absorption enhancement of optical sensing devices based on rippled graphite nanofilm under different stretch:release ratios (0%, 10%, 30%, and 50%).

To confirm our experimental results, a 3D finite-difference time-domain (FDTD) simulation was conducted using Lumerical FDTD Solutions (Release 2018a, Ansys Ltd., Suite, Montreal, QC, Canada) tools. A normal incidence plane wave injection was used. The incident wavelength sweep was from 400 nm to 800 nm. Details of the simulation process can be found in a previous study [37]. A profile monitor was utilized to record the transmitted and reflected fields through the structure. This was followed by the absorption spectra A(λ), which were calculated using the conversion given by A(λ) + R(λ) + T(λ) = 1, where λ is the wavelength and R(λ) and T(λ) are the reflection and transmission spectra, respectively. Figure 5 exhibits the simulated optical behaviors of reflectance, transmittance, absorption, and absorption enhancement spectra of pristine graphite/Si, pristine graphite/PDMS (0%), stretched PDMS/rippled graphite/Si (10%), stretched PDMS/rippled graphite/Si (30%), and stretched PDMS/rippled graphite/Si (50%) structures. As shown in Figure 5A, the reflection decreased when the pristine graphite layer shifted (i.e., 0%, 10%, 30%, and 50%). The lowest values of reflection were observed at wavelengths near the UV spectrum (400 nm), whereas the highest values were recorded at near-infrared (IR) wavelengths (800 nm). This enhancement in reflectivity was attributed to the effect of the roughness patterns in the pristine graphite layer that had been induced by the compressed PDMS layer. Extensive details about the interpretation can be found in our previous work using the specular and multiple internal reflection mechanism [35].

The transmission remained almost the same over the wavelength range of 400–532 nm and gradually increased along the visible spectrum when greater than 532 nm, as shown in Figure 5B. Overall, high transmission occurred in the structures processed with PDMS, as compared to the pristine graphite/Si reference structure. This confirmed that the enhancement of light absorbed was related to the internal reflection process, which led to a reduction in the reflectance and, consequently, an increase in the fraction of power absorbed. Overall, the absorption decreased in broader wavelengths closer to 800 nm, as illustrated in Figure 5C. The absorption was higher in the structures processed with PDMS, as compared to that of the pristine graphite/Si reference structure. This result was in accordance with that reported by Leung et al. [38], where it was established that the absorption depends on parameters such as correlation length and the height of the root mean square of the rough surface, resulting in high absorptivity at higher frequencies. In order to derive a reliable criterion for the enhancement of light absorption, we considered the pristine graphite/Si as a reference structure, and all structures processed with PDMS were compared to the reference structure by a factor named “absorption enhancement” and given by the following equation: $E(\%)=\left[\left(A_{PDMS}-A_{ref}\right)/A_{ref}\right]\times 100$, where *A_PDMS_* and *A_ref_* are the absorptions in the structures processed by PDMS and the reference structure, respectively; the outcome is illustrated in Figure 5D. Stronger enhancement was achieved at longer wavelengths in the visible spectrum and could be controlled by the roughness features. In addition, the stretched PDMS/rippled graphite/Si (30%) had the most enhancement, which, in turn, improved the responsivity of the device and was consistent with the high responsivity of stretched PDMS/rippled graphite/Si (30%), as shown in Figure 4C.

**Figure 6:**
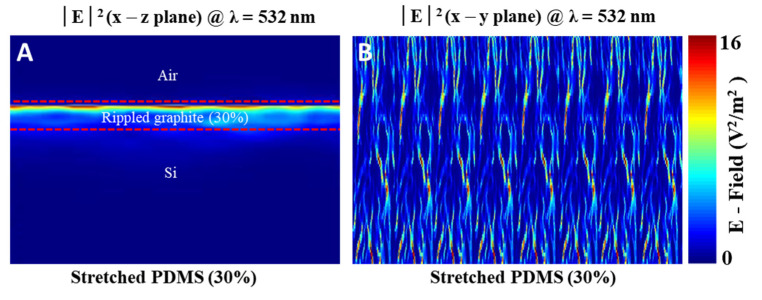
FDTD-simulated electric field intensity distribution of the stretched PDMS/rippled graphite/Si (30%) at 532 nm laser illumination. (**A**) intensity profile at the cross-section through x − z plane, (**B**) intensity profile at the cross-section through x − y plane.

Additionally, the electric field distributions in the x–z and x–y planes were plotted, as shown in Figure 6A,B, at an illumination wavelength of 532 nm. The stretched PDMS/rippled graphite/Si (30%) proved to be the optimum optical sensing device, because it exhibited the highest photoresponsivity value (R = 260 mA/W) (Table 1). A strong response in the near-field intensity distribution was predicted and observed in the high performance of the stretched PDMS/rippled graphite/Si (30%). The result shown in the x–y plane exhibits a similar trend to that of the SEM image depicted in Figure 2C, confirming the accuracy of the numerical simulation in Figure 6A,B.

## 4. Conclusions

By layering graphite nanofilm over a supporting layer of PDMS that could be tested at different stretch:release ratios, we were able to examine whether this enhanced the optical sensing of the rippled graphite surface. We showed that increasing the areal ripple density of the graphite nanofilm surface resulted in enhancement of the optical extinction, which led to enhancements in photoresponsivity. This study showed the potential of enhanced, strain-tunable, and wavelength-specific optoelectronics as well as the enhancement and tuning of optical sensing in future high-performance optoelectronics. Although the optical sensing potential of the rippled graphite nanofilm was enhanced, future improvements concerning the interface contacts between the graphite nanofilm and Si will be essential to obtain a more reliable, stable structure with improved contacts. Future research should involve the exploration of other applications in electronics and optoelectronics in terms of wavelength broadband from X-ray and UV–Vis to IR using other potential nanomaterials (e.g., black phosphorous, hexagonal boron nitride, graphene, MXenes, or transition metal dichalcogenides) that may mimic or improve on this rippled nanofilm surface as well as experimenting with other flexible and/or transparent substrates (e.g., polyethylene terephthalate, polyetherimide, or polyvinylidene fluorine) instead of Si.

## Figures and Tables

**Table 1:** The merit of photoresponsivity and noise-equivalent power (NEP) values of optical sensing devices based on stretched PDMS/rippled graphite at different stretch:release ratios under 532 nm laser illumination.

Stretch–Release Ratio of Graphite/PDMS	0%	10%	30%	50%
Photoresponsivity at the lowest power (100 nW)	20 mA/W	120 mA/W	260 mA/W	13 mA/W
Noise–equivalent power (NEP)	15.74 × 10^−9^ W/(Hz)^1/2^	9.65 × 10^−9^ W/(Hz)^1/2^	7.54 × 10^−9^ W/(Hz)^1/2^	10.21 × 10^−9^ W/(Hz)^1/2^

## Supplementary Materials

The following are available online at https://www.mdpi.com/article/10.3390/nano11112934/s1, Figure S1: A 3D schematic of the stretching apparatus; Figure S2: SEM (A) and AFM (B,C) images of the pristine stretchable PDMS supporting layer; Figure S3: AFM and SEM images of (A–C) pristine graphite/PDMS (0%), (D–F) rippled graphite/stretched PDMS (10%), and (G–I) rippled graphite/stretched PDMS (30%); Figure S4: SEM images at a tilt of 60° of (A) rippled graphite/stretched PDMS (30% as the optimized condition for optical sensing), (B) rippled graphite/stretched PDMS (50% as the upper failure condition).
