# Supplementary material for: The Ripple Effect of Graphite Nanofilm on Stretchable Polydimethylsiloxane for Optical Sensing
Source: Nanomaterials (Basel). 2021 Nov 2;11(11):2934. doi: 10.3390/nano11112934 (PMC8619932; doi:10.3390/nano11112934)
Supplement: Supplementary file 1 [file nanomaterials-11-02934-s001.zip › nanomaterials-1429991-supplementary materials.pdf]

nanomaterials

Supplementary Materials

The Ripple Effect of Graphite Nanofilm on Stretchable Polydimethylsiloxane for Optical Sensing

Kossi A. A. Min-Dianey [1,†], Top Khac Le [2,†], Akeel Qadir [3,4,†], Noé Landry Privace M'Bouana [5,†], Muhammad Malik [6], Sok Won Kim [2,*], Jeong Ryeol Choi [7,*] and Phuong V. Pham [8,*]

1 Département de Physique, Faculté Des Sciences (FDS), Université de Lomé, Lomé 01BP1515, Togo; anyaratt20@yahoo.fr
2 Department of Physics and Energy Harvest Storage Research Center, University of Ulsan, Ulsan 44610, Korea; lekhactop@gmail.com
3 Research Center of Smart Sensing Chips, Ningbo Institute of Northwestern Polytechnical University, Ningbo 315103, China; aki@nwpu.edu.cn
4 Key Laboratory of Micro/Nano Systems for Aerospace (Ministry of Education), and Shaanxi Province Key Laboratory of Micro and Nano Electro-Mechanical Systems, Department of Microsystems Engineering, Northwestern Polytechnical University, Xi'an 710072, China
5 Institut Supérieur de Technologie, Université de Bangui, Bangui BP 892, Central African Republic; mbouana@hotmail.fr
6 Department of Electrical Engineering and Technology, Government College University, Faisalabad 38000, Pakistan; mmalik@gcuf.edu.pk
7 Department of Nanoengineering, Kyonggi University, Suwon 16227, Korea
8 SKKU Advanced Institute of Nano Technology, Sungkyunkwan University, Suwon 440746, Korea
* Correspondence: sokkim@ulsan.ac.kr (S.W.K.); jrchoi@kyonggi.ac.kr (J.R.C.); pvphuong@skku.edu (P.V.P.)
† These authors contributed equally to this work.

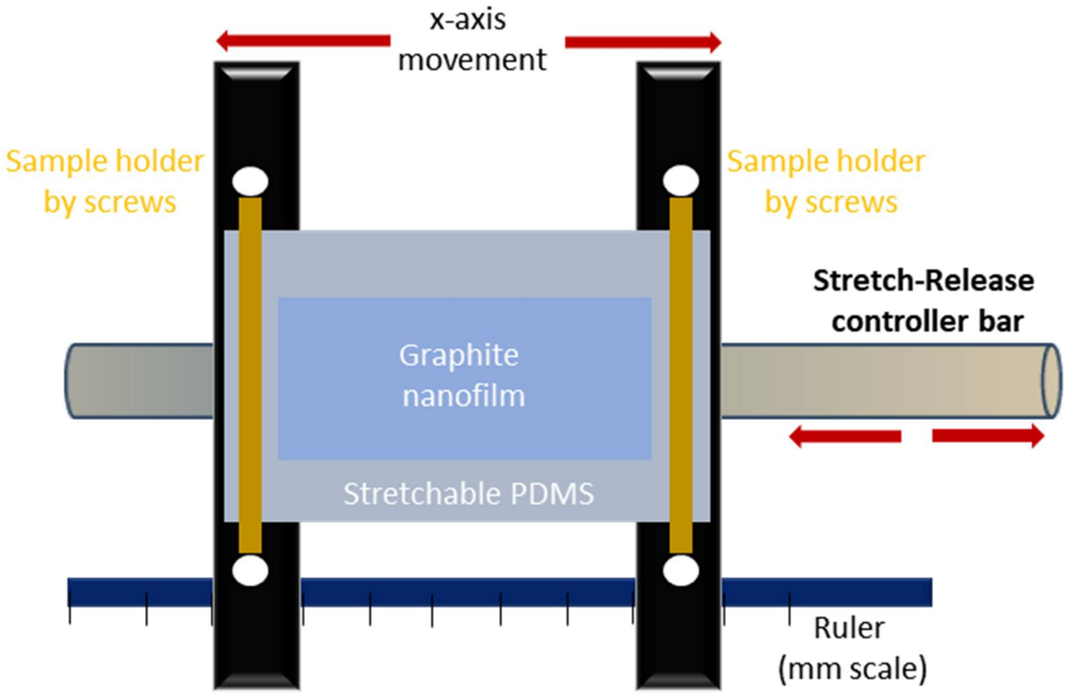

Figure S1. A 3D schematic of stretch-release apparatus.

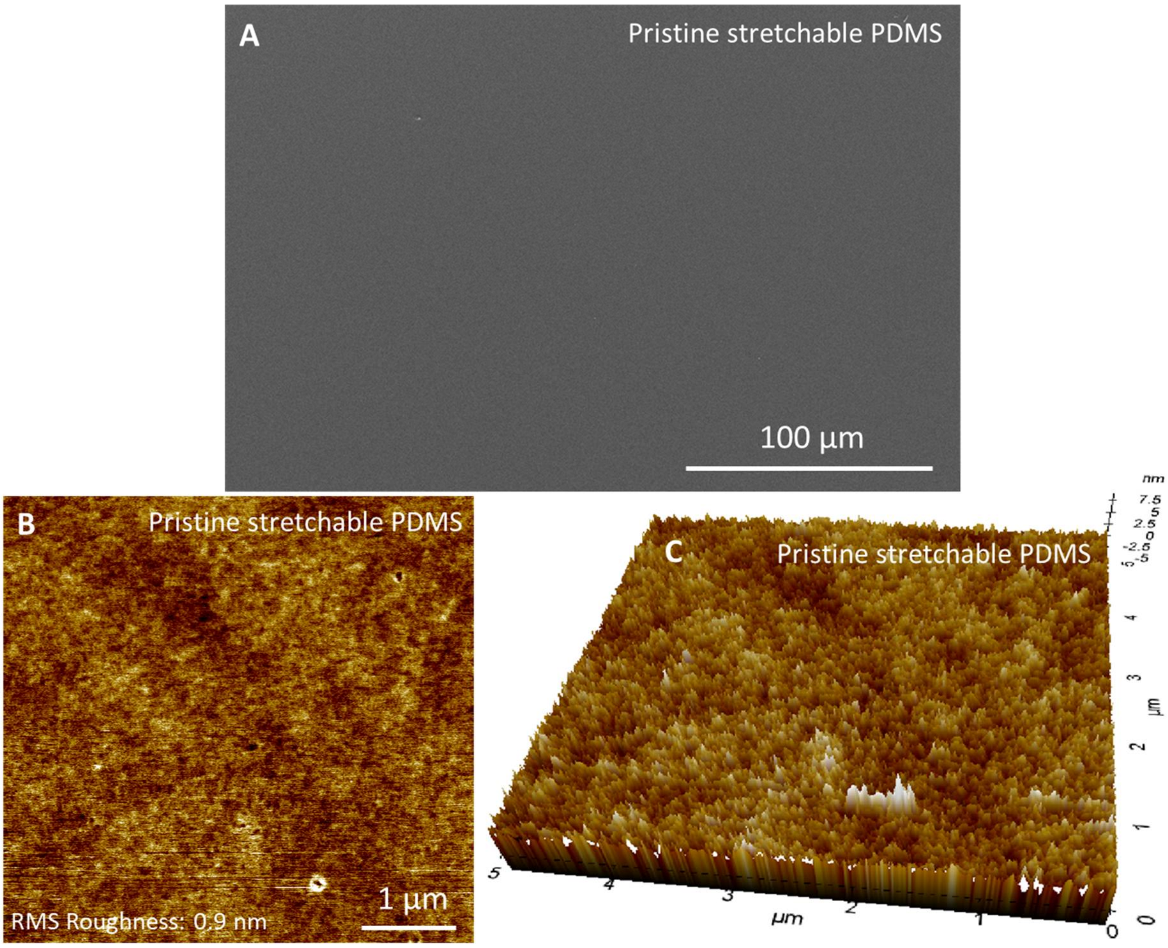

Figure S2. SEM (**A**) and AFM (**B,C**) images of pristine stretchable PDMS supporting layer.

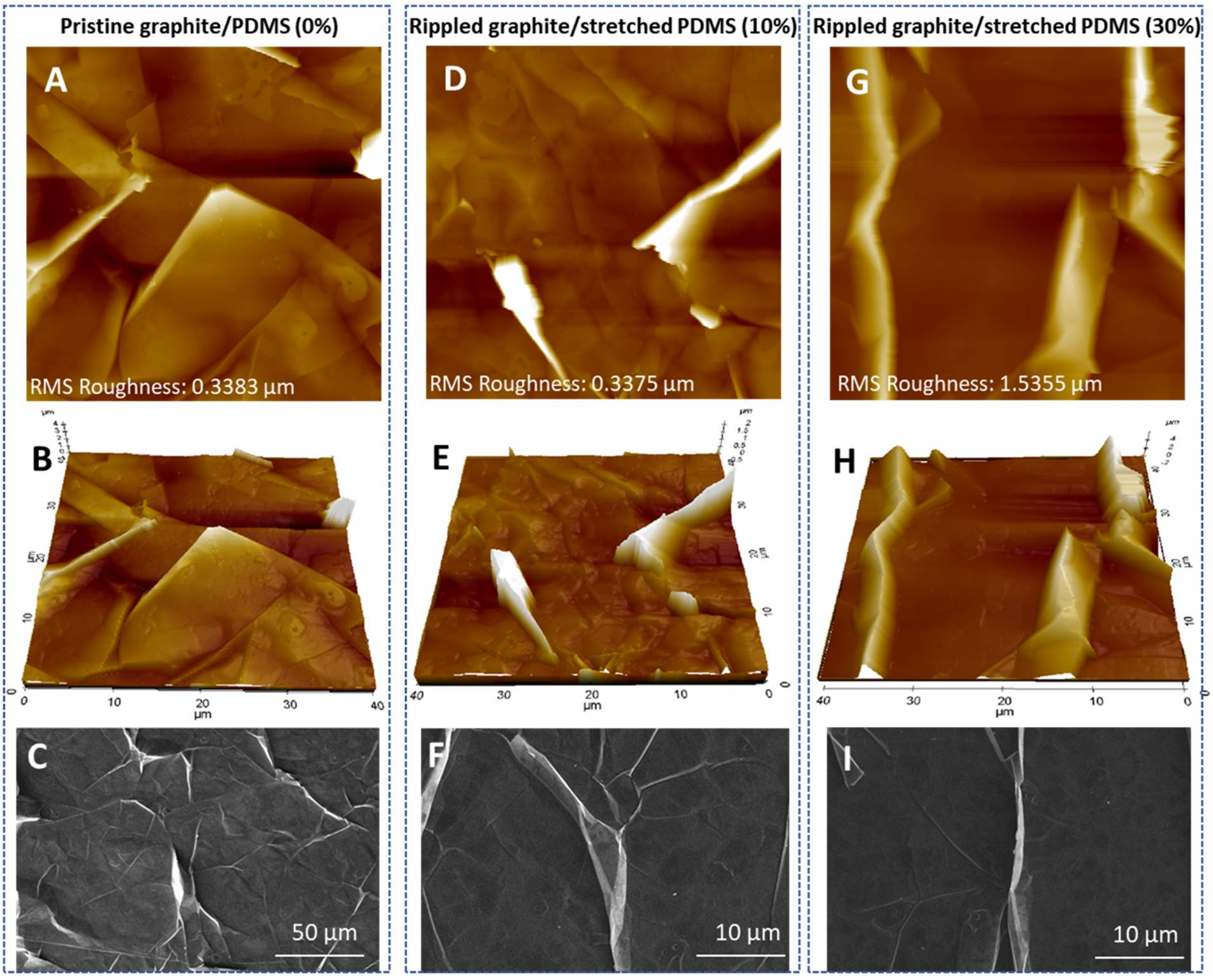

Figure S3. AFM and SEM images of (**A–C**) pristine graphite/PDMS (0%), (**D–F**) rippled graphite/stretched PDMS (10%), and (**G–I**) rippled graphite/stretched PDMS (30%).

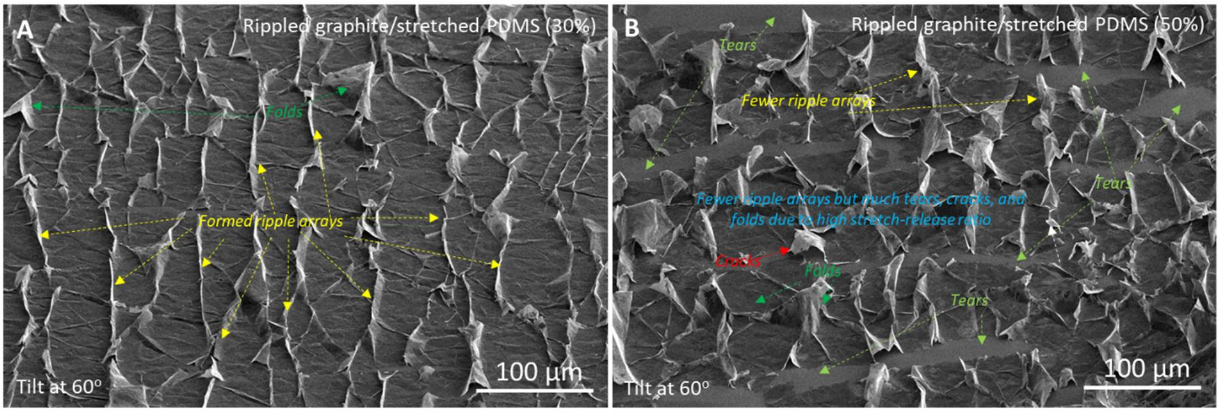

Figure S4. SEM images at a tilt of 60^0 of (**A**) rippled graphite/stretched PDMS (30% as the optimized condition for optical sensing), (**B**) rippled graphite/stretched PDMS (50% as the upper failure condition).
